# The role of autophagy in synucleinopathy: clearance versus spread of α-synuclein

**DOI:** 10.1080/27694127.2025.2577406

**Published:** 2025-10-30

**Authors:** Emily Birnbaum, Zhenyu Yue

**Affiliations:** a Department of Neurology, The Friedman Brain Institute, Icahn School of Medicine at Mount Sinai, New York, NY, USA; b Center for Parkinson’s Disease Neurobiology, Icahn School of Medicine at Mount Sinai, New York, NY, USA

**Keywords:** α-synuclein, autophagy, fibrils, synucleinopathy, Parkinson’s disease

## Abstract

Emerging evidence suggests that the propagation of α-synuclein pathology underlies the progression of Parkinson’s disease and supports the hypothesis that transmission of α-synuclein aggregates contributes to dopaminergic degeneration. Autophagy, a cellular degradation process, removes protein aggregates and damaged organelles and aids in α-synuclein clearance. However, fibrillar α-synuclein aggregates may evade and even disrupt autophagy, causing toxic spread. The role of autophagy may be multifaceted in the propagation of α-synuclein: clearing α-synuclein aggregates and damaged organelles (protective) versus the release of α-synuclein aggregates (harmful). Here we review how neuronal and glial autophagy regulate α-synuclein clearance and spreading. We also discuss the need for future research to address the interplay of autophagy and α-synuclein aggregates toward therapeutic development.

## Introduction

Synucleinopathies are a group of neurodegenerative diseases characterized by α-synuclein inclusions in the central nervous system (CNS). They include Parkinson’s Disease (PD), Multiple System Atrophy (MSA), and Dementia with Lewy bodies (DLB). The main pathologies of PD include the accumulation of α-synuclein and death of dopaminergic neurons in the substantia nigra (SN)^[1]^. α-synuclein is a small protein that is intrinsically disordered. It is prone to misfolding and aggregation leading to oligomerization, fibrilization, and accumulation into inclusions called Lewy bodies (LBs)^[2,3]^. Additionally, α-synuclein can propagate in a prion-like manner between cells, including neurons and glia, and seed into new cells leading to the conversion of endogenous monomeric α-synuclein into pathological aggregates^[1]^. While α-synuclein can be cleared by autophagy, several studies have shown that α-synuclein expression and aggregation impairs autophagic flux^[4–6]^. Different forms of α-synuclein, especially the fibrillar form can evade autophagy leading to increased toxicity and neuronal cell death^[7]^. However, the details for how exactly different forms of α-synuclein intersect with autophagy pathways remains poorly understood. The uncharacterized mechanism whereby α-synuclein fibrils escape or even impair autophagy hinders therapeutic development. Gaining a better understanding of the molecular mechanism is crucial for identification of novel therapeutic strategies targeting autophagy to treat synucleinopathies.

In this review we provide an overview of the interplay between α-synuclein and autophagy process. We review the evidence that autophagy degrades α-synuclein and how fibrillar α-synuclein may evade and impair autophagy in disease. We also highlight the need for future research to address these mechanisms toward therapeutic development.

## Autophagy pathways and the role of autophagy in the CNS

The autophagy system consists of a set of pathways in which cytoplasmic material is delivered to lysosomes for degradation. These pathways include microautophagy, chaperone-mediated autophagy (CMA), and macroautophagy/autophagy which will be the focus of this review^[8,9]^. While microautophagy and CMA directly deliver substrates to the lysosome, autophagy requires the formation of a vesicle called an autophagosome which delivers cargo by fusing to a lysosome^[9,10]^. Autophagy includes five phases: induction, elongation, cargo recognition, maturation and transport, and fusion with lysosomes^[11]^.

Initiation of autophagy can be either starvation-induced or driven by specific cargo, both of which involve the unc-51 like autophagy activating kinase (ULK) complex which consists of the scaffold protein FAK-family interacting protein 200 (FIP200), specific autophagy-related genes ATG101 and ATG13, and the serine/threonine kinase ULK1 or ULK2^[11,12]^. ULK activity is suppressed by mammalian target of rapamycin 1 (mTORC1), which is inactivated in the context of starvation leading to activation and assembly of the ULK complex that recruits ATG proteins resulting in autophagosome formation. ULK complex activity can also be triggered by selective autophagy cargo receptors such as SQSTM1/p62 (sequestosome 1). Cargo receptors form condensates with ubiquitinated proteins and interact with FIP200, thus recruiting the ULK complex^[13]^. The ULK complex then recruits the nucleation complex consisting of Beclin 1, VPS34, VPS15, ATG14, and nuclear receptor binding factor 2 (NRBF2) to the membrane, which generates phosphatidynlinositol-3-phosphate (PI3P) leading to elongation of the autophagosome membrane^[14,15]^. Additionally, at this phase, ATG9 vesicles are recruited to the membrane by ATG2 and WD repeat domain phosphoinositide interacting 2 (WIPI2), playing a key role in lipid transfer^[14]^. In selective autophagy, autophagy receptors recognize cargos tagged with degradation signals such as ubiquitin and connect them to the autophagosome membrane via its LC3-interacting regions^[16,17]^. Closure is mediated by the Endosomal sorting complexes required for transport (ESCRT) complex leading to formation of the autophagosome. The fully formed autophagosome is recruited to dynein by scaffolding proteins and transported to a lysosome rich region^[18,19]^. Finally, mediated by SNAP receptor (SNARE) protein complexes and the HOPS complex, autophagosomes fuse to lysosomes to form the autolysosome, leading to degradation^[20]^.

Autophagy plays a key role in maintaining homeostasis in the CNS, and its dysfunction contributes to neurological disorders including neurodegenerative diseases. Cells of the CNS have significant metabolic and catabolic demands such as the need to recycle proteins, lipids, and organelles^[21]^. The autophagy-lysosomal pathway (ALP) can meet these needs. Autophagy maintains neuronal homeostasis via constant digestion and recycling of neuronal materials^[22–25]^. Numerous studies focused on autophagy cargo profiling have demonstrated the selective degradation of organelles and protein complexes in neurons^[23,26–28]^. By isolating and purifying autophagosomes, researchers have identified specific pathways involved in ER-phagy, golgi-phagy mitophagy, and aggrephagy via distinct autophagy receptors^[27,28]^. Autophagy also plays a key role in the regulation of synaptic activity and synaptic vesicle trafficking^[22,26]^. Notably, synaptic vesicle proteins were highly enriched in neuronal autophagy cargo^[28]^. Autophagy has been shown to affect synaptic transmission by controlling neuronal excitability through regulating levels of specific channel proteins including the calcium efflux channel ryanodine receptor 2 (RyR2) and the Kir2 potassium channel^[26,29]^. Moreover, autophagy regulates phosphokinase A (PKA) type I complex homeostasis and activity through autophagy receptor A-kinase anchoring protein 11 (AKAP11), controlling synaptic transmission^[24]^. In addition to neurons, autophagy maintains homeostasis in glial cells including microglia, astrocytes, and oligodendrocytes in basal conditions and during development^[30–34]^. Microglial autophagy is involved in regulation of the immune response. For example, autophagy in microglia blocks NLR-family-pyrin-domain-containing 3 (NLRP3) inflammasome activation in addition to lipopolysaccharide (LPS)-induced inflammatory responses^[35,36]^. Astrocytic autophagy is critical for astrocyte differentiation and plays a key role in their mitochondrial maintenance and survival^[37,38]^. Additionally, autophagy plays a vital role in promoting and regulating myelination. Suppression of Atg7 in mice led to age-dependent myelin accumulation, increased sheath thickness, and abnormal myelin structure^[30,39]^. Overall, degradation by autophagy is required for many vital processes in the CNS across multiple cell types.

## α-synuclein

### Structure and function

α-synuclein, produced by *SCNA* gene, is highly expressed in neurons, and its 140 amino acid sequence is divided into three domains: (1) the N-terminal domain (residues 1–60), (2) the non-amyloid-Β-component (NAC) central region (residues 61–95), and (3) the C-terminal domain (residues 96–140). The N-terminal domain is amphiphilic and can bind to phospholipid vesicles and the plasma membrane^[40]^. Point mutations in this region, such as A53T and E47K, are associated with autosomal dominant forms of familial PD and lead to the formation of α-synuclein fibrils, while A30P causes a predisposition to α-synuclein oligomer aggregation^[41,42]^. The NAC domain is hydrophobic and important for α-synuclein aggregation^[40]^. This domain can form β-sheet structures^[43]^. The C-terminal domain is enriched with acidic amino acids and can flexibly adapt to different structures^[44]^. When α-synuclein N-terminal domain binds to lipids or lipid membranes, it forms an increased α-helical structure which can actually inhibit fibril formation^[45,46]^. The biological function of α-synuclein is not fully defined; however studies suggest its roles in synaptic plasticity and synaptic vesicle release in addition to its contribution to PD pathology^[47,48]^.

### Misfolding and aggregation

In PD cells, misfolding of α-synuclein leads to the formation of oligomers from monomers. These oligomers have an antiparallel β-sheet structure^[49]^. Oligomer complexes are formed by non-covalent protein binding. In the case of α-synuclein, these oligomers are highly heterogeneous containing few to dozens of monomers. During aggregation, some oligomers form fibrils, known as “on-pathway” species, while other oligomers that cannot form fibrils are termed “off-pathway” species^[50]^. While the exact structure of α-synuclein oligomers is not fully known, cryo-electron microscopy (EM) studies have revealed the structure of two subgroups. Both have hollow cylindrical structures^[51,52]^. Certain oligomeric forms of α-synuclein can enter cultured cells and promote aggregation of cytosolic α-synuclein, demonstrating a seeding effect. One study showed that extracellular α-synuclein oligomers can cross cell membrane and promote intracellular oligomerization^[53]^.

The “on-pathway” oligomers form β-sheet rich protofibrils leading to the formation of compact insoluble fibrils^[49]^. Nuclear magnetic resonance (NMR) studies suggest that the fibril core contains the NAC region and part of the N-terminal domain, assembled into five to six β strands separated by loops. The rest of the N-terminal region and the C-terminal regions appear unstructured and are outside of the core^[54]^. Electron microscopy and atomic force microscopy are used to characterize the morphology of fibrils, and various strains have been reported. Multiple studies have demonstrated that this structural variability is due to the conditions in which they assemble^[55–57]^. Additionally, since fibril formation is sensitive to changes in the microenvironment of the protein, single amino acid changes can result in different polymorphisms due to site-specific conformational dynamics. This has been demonstrated with familial Parkinson’s disease mutations^[58]^. Several distinct polymorphs have been reported from cryo-EM studies^[59]^. The structure of pathological α-synuclein filaments varies based on the specific synucleinopathies. Lewy body diseases including PD and DLB have the same protofilament fold, while MSA cases are represented by two distinct filament types each formed by two protofilaments^[60,61]^. There is a need for in vitro and in vivo correlations with these different fibrillar species to understand biological and clinical implications of polymorphic variability.

### Post-translational modifications

α-synuclein is susceptible to multiple post-translational modifications (PTMs) that can affect its folding and conformation, resulting in changes in its physiological and pathological function^[59,62]^. Phosphorylation can affect the structural conformation of α-synuclein^[63]^. Phosphorylation at serine residues 129 and 87 is elevated in LB compared to healthy brain^[49]^. Some studies have shown that phosphorylation at S129 accelerates the formation of inclusions and toxicity in cell models^[64,65]^. Howecer, recent studies have suggested S129 phosphorylation is secondary to α-synuclein fibril formation and may have a protective effect by inhibiting further aggregation^[66,67]^. Interestingly, S129 phosphorylation has also been shown to promote autophagic clearance of α-synuclein aggregates. A similar effect on autophagy is seen with phosphorylation or nitration at Y133^[67]^. Effects are dependent on the phosphorylation site with other sites having distinct effects including modulating the amplification and seeding capacity^[68]^. Ubiquitination of α-synuclein generally promotes it’s uptake for autophagic or proteasomal degradation depending on the site and ligase^[69]^. N-terminal acetylation of α-synuclein leads to stronger membrane affinity because it can cause formation of α-helical structure on the N-terminus. This modification reduces the aggregation capability of α-synuclein^[70]^. SUMOylation involves the covalent attachment of the small ubiquitin-like modifier (SUMO)^[71]^. The addition of SUMO protein, mediated by the E3 ligase PIAS2 (protein inhibitor of activated STAT2), can lead to accumulation of α-synuclein. SUMOylation has been shown to impair α-synuclein ubiquitination, thus promoting aggregation^[72,73]^. Other studies have displayed a protective role of SUMOylation. Specifically, SUMOylated α-synuclein showed reduced in vitro fibril formation as compared to unmodified α-synuclein. Further, substitutions of lysine 96 and 102 to arginine on α-synuclein impaired SUMOylation and resulted in enhanced aggregation in substantia nigra neurons in a PD rat model, further highlighting this protective effect^[74]^. The C-terminal domain of α-synuclein has protective features, such as motifs for ubiquitin ligases and autophagy mediators, that limit misfolding and aggregation and promote degradation. As such, when the region is truncated, aggregation increases and degradation is reduced^[75,76]^. The loss of more than 15 residues of this domain through truncation has been shown to promote accelerated formation of fibrils and lead to more twisted and shorter fragmented fibrils^[75]^. O-GlcNAcylation is a modification of serine and threonine residues. Specifically, it involves the addition of N-acetylglucosamine by O-GlcNAc transferase (OGT)^[77]^. O-GlcNAcylation at residues T72, T75, T81 and S87 inhibits aggregation of α-synuclein monomers into fibrils; however, this inhibition may promote toxic oligomer formation^[77]^. Overall, PTMs of α-synuclein can affect the spreading of pathological α-synuclein by modifying aggregation, seeding capacities, and degradation (Table 1).

**Table 1.** Common post-translational modifications of α-Synuclein.

Modification	Residue/Location	Physiological Implications
Phosphorylation	S129	Accelerate formation of inclusions and toxicity Inhibit aggregation Promote autophagy
	Y133	Promote autophagy
	Various sites	Modulate amplification and seeding capacity
Nitration	Y133	Promote autophagy
Ubiquitination	Various sites	Promote autophagy or proteasome degradation
N-terminal Acetylation	N-terminus	Reduce aggregation
SUMOylation	K102, K96	Inhibit aggregation and prevent toxicity
C-terminal Trunction	C-terminus	Increased misfolding, aggregation, and fibril formation Reduced autophagy
O-GlcNAcylation	T72, T75, T81	Inhibit aggregation Promote oligomer formation

### Clinical applications and diagnostics

The development of new methods to detect pathological α-synuclein including seed-amplifications assays (SAAs) and brain imaging techniques will improve the diagnosis of synucleinopathies as well as lead to a better understanding of pathology. SAAs utilize protein misfolding cyclic amplification to allow for the detection of the seeding capacity of any α-synuclein present in biological samples^[78]^. The assay specifically exploits pathological propagation of α-synuclein species to multiply them in vitro. This technique allows for improved and more sensitive diagnostics while also enabling monitoring of the kinetics of α-synuclein aggregation and its biophysical properties^[79]^. Multiple PET ligands are currently in development for imaging of α-synuclein aggregates. One such ligand is the F0502B PET tracer that recognizes α-synuclein in mice, non-human primates, and humans and binds with high affinity, however more studies are required to test potential off-target effects^[80]^. Another ligand, the ACI-12589 PET tracer, is specific for pathological α-synuclein in tissue from people with multiple system atrophy (MSA). Only limited binding was seen in both PD and DLB without a definitive explanation, suggesting a need for additional studies in non-MSA cases^[81]^. Additionally, post-translational modifications in vivo may lead to structural changes, leading to a variation in binding potency of PET ligands^[82]^. PET tracers may also be suitable to demonstrate conformations and biophysical properties of various α-synuclein strains in addition to tracing its spread^[79]^.

Given the cascade of α-synuclein misfolding and aggregation and variety of strains of the protein, there are many intervention points at which it could be targeted as a therapeutic strategy in PD and other synucleinopathies. One point of intervention would be to target autophagy clearance specifically. Certain tools are being studied such as the autophagosome-anchoring chimera that can enhance binding of α-synuclein to autophagic machinery, thus inducing degradation^[83]^. Additional studies aim to utilize small molecules to activate autophagy, however they generally do not address clearance of α-synuclein aggregates^[84]^. More knowledge on mechanisms by which aggregated forms disrupt autophagy will improve our ability to target autophagy therapeutically. Here, we will focus on clearance mechanisms of α-synuclein. Specifically, we review the process of autophagy of α-synuclein and how it is disrupted in pathological states.

## Degradation of α-synuclein by autophagy in the CNS

### Homeostatic α-synuclein clearance in neurons

Earlier evidence indicated that α-synuclein is degraded by the ubiquitin-proteasome system (UPS), chaperone-mediated autophagy (CMA), and macroautophagy^[85,86]^. However, in its aggregated form, α-synuclein cannot be degraded by UPS but instead impairs the system, in which case autophagy plays a key role^[56,87–89]^. Interestingly, the WT α-synuclein protein sequence harbors the CMA-recognition motif VKKDQ, and mutations in this motif lead to a reduction of α-synuclein translocation to the lysosome^[90]^. The recognition motif specifically allows the protein to be recognized by Heat shock cognate protein 70 (HSC70) and bind to Lysosome-associated membrane protein 2A (LAMP2A) at the lysosomal membrane. However, A53T and A30P α-synuclein mutants block uptake by binding to LAMP2A receptors on the lysosomal membrane but fail to cross the membrane, thus blocking CMA^[91]^. Other mutations and post-translational modifications can impair degradation by CMA without blocking it completely, resulting in α-synuclein inclusions in the brain^[86]^. Constitutive deletion of essential autophagy gene *Atg7* in dopamine neurons of mice only causes an age-dependent accumulation of α-synuclein at presynaptic terminals without apparent inclusions in the soma, suggesting a non-critical role of autophagy in clearing endogenous α-synuclein in young adult neurons, but pointing to a function of autophagy in regulating α-synuclein homeostasis in aged neurons^[92]^. As such, autophagy is crucial for degrading pathologic α-synuclein species, which are associated with aged neurons^[93,94]^.

### Microglial uptake and clearance of α-synuclein via synucleinphagy

Multiple studies have suggested that Toll-like receptors (TLRs), such as TLR2 and TLR4, can act as receptors for soluble α-synuclein^[95–99]^. TLR4 is required for microglial signaling through interaction with α-synuclein leading to autophagy but may also induce neuroinflammation and cell to cell transmission^[100]^. One study demonstrated that TLR4 ablation impaired microglial phagocytosis of α-synuclein and increased neurodegeneration in a mouse model of MSA^[101]^. However, other studies have shown that α-synuclein induces secretion of inflammatory cytokines including interleukin-1β (IL-1β) and tumor necrosis factor-α (TNF-α) through TLR4 interaction^[102]^. We showed that neuron-released α-synuclein triggered microglial TLR-4 signaling, which induces selective autophagy degradation of internalized α-synuclein specifically in microglia^[95]^. The study also suggests that extracellular α-synuclein interacts with TLR-4, triggering nuclear factor-κB (NF-κB) signaling leading to p62 induction and autophagy but without inducing type I interferon response. Our study demonstrates a critical role for microglial autophagy in maintaining α-synuclein homeostasis in the brains and preventing synucleinopathy and neurodegeneration^[95]^.

### Cargo recognition and binding to autophagic machinery

The internalized α-synuclein in is labeled with ubiquitin, which is known to be recognized by p62, which could explain the selective degradation of α-synuclein through autophagy^[95]^. But it remains unclear exactly how α-synuclein may be ubiquitinated^[17]^ and if the p62 binding is sufficient to mediate autophagy. One study found that NEMO (nuclear factor-κB essential modulator) acts as an autophagy adapter protein for α-synuclein. Specifically, they found enrichment of both M1-linked ubiquitin and NEMO at α-synuclein aggregates in a cellular seeding model in SY5Y cells. Further, they showed that binding of NEMO to M1-linked ubiquitin created a phase segregated aggregate surface that co-condenses with p62, aiding in the efficient recruitment of autophagic machinery^[103]^. A few potential E3 ligases for α-synuclein have been reported^[69]^. Specifically, both Neuronal precursor cells expressed developmentally downregulated 4 (NEDD-4) and Listerin E3 ligases polyubiquitinate α-synuclein leading to endosomal degradation^[104,105]^. Additionally, Siah-2, a RING-type E3 ligase, has been shown to ubiquitinate α-synuclein at several sites leading to degradation^[69]^. Future studies should aim to better identify the binding mechanism of p62 and NEMO for different forms of α-synuclein and different cell types in the CNS, and whether other proteins or specific ubiquitin ligases are involved.

## α-synuclein intercellular transmission and disruption of autophagy in Parkinson’s disease

### Intercellular transmission of α-synuclein

Early studies established that α-synuclein can be transferred between cells even before the Preformed Fibril (PFF) model was developed. Intercellular transmission was first suggested when Lewy bodies were discovered in fetal mesencephalic neurons grafted into the striatum of PD patients, indicating host-to-graft propagation of α-synuclein pathology^[106]^. This finding prompted investigation into mechanisms of cell-to-cell transfer, revealing that neurons can release α-synuclein through both conventional and unconventional secretory pathways. Under physiological conditions, a small portion of cellular α-synuclein is secreted via exocytosis, with increased secretion occurring during proteasomal and mitochondrial dysfunction^[106]^. The secreted α-synuclein can be monomeric or oligomeric, with oligomeric forms being more prone to propagation. Low-density lipoprotein receptor-related protein (LRP1) has been found to mediate uptake of α-synuclein in its monomeric, oligomeric, and fibrillar forms in neurons. This extracellular α-synuclein can be taken up by neighboring cells through various mechanisms including endocytosis, potentially initiating new aggregation seeds in recipient cells^[107]^.

The pathological spread hypothesis gained substantial support with the development of the PFF model, where exogenous introduction of synthetic α-synuclein fibrils demonstrated cell-to-cell transmission capabilities. Intrastriatal injection of PFFs into mouse brains causes Lewy body-like pathology and spreading across interconnected brain regions^[108]^, establishing a mechanistic link between protein propagation and disease progression. The PFF model has since become central to understanding α-synuclein pathology^[107]^. Primary cortical neurons can internalize these fibrils through receptor-mediated endocytosis, with evidence showing anterograde axonal transport of fibrils to recipient neurons^[109]^. The pS129 modification can enhance interaction between fibrils and receptors, increasing cellular internalization and seeding potential^[110]^. Multiple receptors have been identified that facilitate fibril uptake, including lymphocyte activation gene 3 (LAG3), amyloid precursor-like protein 1 (APLP1) and Family with sequence similarity 171 (FAM171A2)^[110–112]^ which recognize and bind to fibrils promoting uptake primarily in neurons, highlighting potential therapeutic targets for disrupting transmission^[113]^. Additionally, heparan sulfate proteoglycans (HSPGs) mediate fibrillar α-synuclein transmission between neurons^[114]^.

Microglia play a crucial role in α-synuclein transmission dynamics, with PFF injection inducing brain propagation correlated with microglial activation^[115]^. Recent studies have identified that microglia can both clear and propagate α-synuclein pathology, depending on their activation state^[107]^. Oligomeric α-synuclein interacts with TLR2 leading to internalization, while α-synuclein fibrils interact with FcγRIIB in both microglia and neurons^[116]^. Importantly, when the clearance capacity of microglia is overwhelmed, they may contribute to pathology spread through exosomal release mechanisms^[113]^. Astrocytes similarly participate in α-synuclein homeostasis. While α-synuclein released from neurons can trigger neuroinflammatory responses in astrocytes^[117]^, coculture studies demonstrate that astrocytes can reduce neuronal α-synuclein accumulation through enhanced internalization and degradation capabilities^[118]^. However, α-synuclein inclusions have been found in astrocytes of PD, DLB, and MSA postmortem brains, though these inclusions have a unique ultrastructure compared to neuronal inclusions^[119–121]^. Oligodendrocytes can also participate in α-synuclein internalization as shown in both oligodendroglial cell lines and in vivo when injected into mice^[122,123]^. The gap junction protein connexin-32 (Cx32) plays a role in oligomeric α-synuclein uptake in both neurons and oligodendrocytes^[124]^.

Tunneling nanotubes (TNTs) represent another potential transmission route, functioning as F-actin containing membrane channels that directly connect cells^[125]^. These structures facilitate transfer of α-synuclein between neurons and microglia as well as between astrocytes and microglia^[125–128]^, with recent evidence suggesting they may serve both pathological and protective functions. While TNTs can spread α-synuclein pathology, they may also distribute α-synuclein burden across multiple cells to enhance clearance efficiency^[113,127,128]^. The PFF model has revealed important connections between autophagy impairment and increased α-synuclein exosomal secretion^[107,129]^, suggesting that defective cellular clearance mechanisms may accelerate transmission and disease progression through multiple cell types. This will be discussed in more detail in the following section.

### Unconventional secretion and α-synuclein transmission

The intersection of autophagy dysfunction and α-synuclein secretion may represent a critical mechanism in Parkinson’s disease pathogenesis, where impaired cellular clearance pathways facilitate protein transmission between cells. While canonical autophagy typically degrades cytoplasmic contents through lysosomal fusion, disruption of autophagy can redirect α-synuclein toward unconventional secretory routes, including exophagy (secretion from an autophagy intermediate) and alternative release mechanisms that bypass the classical pathway^[130]^.

Multiple cellular mechanisms contribute to autophagy-mediated α-synuclein secretion. Tubulin polymerization-promoting protein (TPPP/p25α) exemplifies how specific molecular players can hijack autophagic machinery, impairing autophagosome-lysosome fusion and promoting exophagy-mediated α-synuclein release^[131]^. Neuronal activity triggers secretory autophagy, a process demonstrated when glutamate stimulation induces calcium-dependent α-synuclein secretion. During this process, α-synuclein colocalizes with autophagy markers but not with LAMP1, indicating that the protein uses an alternative trafficking pathway that bypasses lysosomal degradation compartments. This neuronal stimulation results in α-synuclein being released both via exosomes and as free protein into the extracellular space^[132]^.

Post-translational modifications further modulate these secretory processes. S-nitrosylation of p62, an autophagy receptor protein, inhibits autophagic flux while simultaneously promoting α-synuclein secretion and intercellular spread^[133]^. This modification, observed in Parkinson’s disease and Lewy body dementia patients, demonstrates how cellular stress responses can convert protective autophagy into pathogenic secretion mechanisms. The resulting α-synuclein release occurs through both extracellular vesicle (EV)-dependent and EV-independent pathways, with EV-mediated transmission being particularly important for cell-to-cell propagation despite representing a smaller fraction of total protein secretion^[133]^.

The LRRK2-Rab10 pathway provides another regulatory axis for α-synuclein secretion from macrophage and microglial cells. Lysosomal stress, particularly following preformed fibril treatment, drives Leucine rich repeat kinase 2 (LRRK2) and Rab10 accumulation on lysosomal surfaces, leading to Rab10 phosphorylation and subsequent exosomal α-synuclein release. This mechanism is particularly relevant as it produces seed-competent α-syn species capable of propagating pathology, highlighting the role of lysosomal dysfunction in generating transmissible pathogenic forms^[134]^.

The pathological consequences extend beyond pathological protein release. Autophagy impairment generates a hostile microenvironment through the secretion of toxic α-synuclein species that induce local neurotoxicity, evidenced by increased caspase-3 positive cells in surrounding tissue. Importantly, the toxicity appears more closely related to released oligomeric species rather than higher-order aggregates. Overexpression of this oligomeric α-synuclein leads to increased CD63 and Rab11a expression suggesting multivesicular body-mediated exosomal release^[135]^.

Lysosomal membrane damage represents another key mechanism where galectin-3 (LGALS3) mediates unconventional α-synuclein secretion via lysosomal exocytosis in human midbrain dopaminergic neurons. When lysosomes containing α-synuclein sustain membrane damage, LGALS3 recruitment promotes vesicle secretion through the autophagy-lysosomal pathway, providing a damage-response mechanism leading to pathogenic protein secretion and transmission^[136]^.

An interesting common thread across many of these studies is that while only a small fraction of secreted α-synuclein is associated with exosomes, levels of exosomal α-synuclein are shown to be more seed-competent and actually correlate with severity of motor dysfunction in PD^[129,132,133]^. Serum-derived exosomes from PD patients led to aggregation and phenotypic PD in mice^[137]^. Specifically, α-synuclein oligomers can be associated with exosomes or free, while exosome associated oligomers are more likely to be taken up and are more toxic to the cell. Autophagy enhancement led to a decrease in oligomer signal in the exosomal fraction of H4 cells and the opposite was observed when autophagosome lysosome fusion was inhibited. The evidence suggests that a larger pool of autophagosomes enhances exosomal release, while enhanced lysosomal activity reduces oligomer secretion^[138]^. Interestingly, there is no obvious morphology difference between α synuclein fibrils formed with or without exosomes, highlighting that the existence of the exosome and possibly its lipid or protein components play an important role^[139]^. Further studies are needed to elucidate the aspects of exosomes that contribute to the progression of pathology and whether some modification of exosomes could affect their spread and toxicity. Collectively, these findings suggest that autophagy dysfunction in neurodegeneration creates a pathological switch where protective clearance mechanisms become mediators of disease spread.

### Autophagy and fibrillar α-synuclein

Fibrillar α-synuclein has been shown to initiate autophagy, which, however, is often unsuccessful in leading to degradation and may contribute to the spread of pathology. Multiple studies and continued research aim at tracking this process to better understand the autophagic machinery involved and the points at which it may be disrupted.

One study found that α-synuclein fibrils are quickly internalized by microglia cells and localized to lysosomes, followed by an increased autophagic response, evidenced by LC3-I to LC3-II conversion and LC3 vesicle formation around α-synuclein-positive lysosomes. This autophagic response coincides precisely with Tank-binding kinase 1 (TBK1) and Optineurin (OPTN) phosphorylation and their colocalization with ubiquitin, LC3, and α-synuclein-positive vesicles, while p62 was notably absent^[140]^. Overall, this study shows that while fibrillar α-synuclein is quickly internalized by lysosomes, it initiates lysosomal damage and recruitment of TBK1/OPTN at the same timepoint in which autophagy is activated. They point to a clear involvement of TBK1 and OPTN of autophagy receptors but raise an important question regarding the actual trigger of canonical autophagy in the context of fibrillar α-synuclein^[140]^.

Another study identified a mechanism for α-synuclein fibril clearance where microglial cells transfer large aggregates to cells with less α-synuclein burden via F-actin positive tunneling nanotubules. This intercellular sharing, also associated with pathological spread, actually appears protective by downregulating inflammatory responses and enabling joint degradation of aggregates for improved clearance^[127]^. It would be interesting to determine if tunneling nanotube transfer of α-synuclein can play a protective role across other cell types. Additionally, this process may represent a target by which we could intervene and utilize the intercellular spreading of α-synuclein to improve fibril clearance in pathological conditions.

### Potential mechanisms for how fibrillar α-synuclein evades and disrupts autophagy-lysosomal pathway

Several studies have shown that α-synuclein fibrils can suppress autophagy and proposed potential mechanisms relating to its interaction with autophagic machinery across different steps of the process. Oligomeric intermediates are generally more susceptible to clearance; however, their aggregation contributes to toxicity and the formation of fibrils. It is also possible that these intermediates can create an excessive burden on clearance machinery^[141]^. Additionally, numerous studies focus on how α-synuclein can suppress autophagy by utilizing α-synuclein overexpression and mutants rather than PFFs to model pathology. The form of α-synuclein used in each study discussed will be specifically noted.

**Figure 1.**
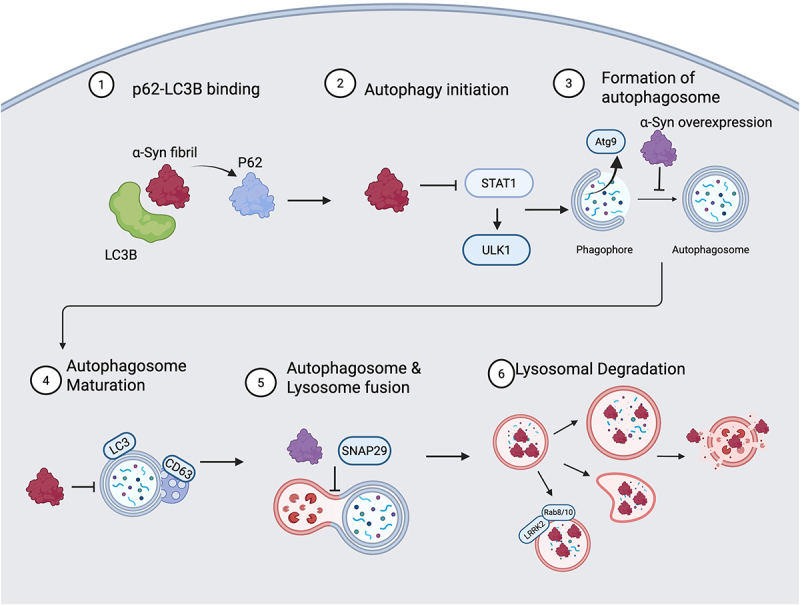
α-synuclein disrupts the autophagy lysosome pathway. α-synuclein fibrils bind to LC3B at a similar site as p62 thus blocking LC3B-p62 binding and suppressing p62-dependent selective autophagy (1). PFFs can reduce autophagy initiation by reducing Stat1 translation of ULK1 (2). α-synuclein overexpression can block the formation of the autophagosome, possibly by causing the displacement of Atg9 which is crucial to autophagosome biogenesis (3). α-synuclein fibrils can suppress autophagosome maturation by disrupting fusion with MVBs (4). α-synuclein overexpression can disrupt the SNARE complex, possibly by reducing SNAP29 leading to failed autophagosome lysosome fusion (5). PFFs have been shown to disrupt lysosomal degradation by altering lysosome structure and reducing lysosomal hydrolase activity. This disruption likely involves the recruitment of LRRK2 and its rab substrates and leads to lysosomal membrane permeability and release of fibrils (6). Created in biorender.

Early studies provided insights into how overexpression of α-synuclein protein disrupts autophagy at multiple steps. One study found that α-synuclein overexpression caused relocation of Atg9, a transmembrane autophagy protein that plays a key role in autophagosome biogenesis. Another study showed that A30P mutant α-synuclein overexpression inhibited autophagy via disruption of autophagosome formation in neurons^[142]^. Both findings support the idea that α-synuclein suppresses autophagy by impairing formation of the autophagosome (Figure 1)^[143]^.

Another point at which α-synuclein likely disrupts autophagy is at the fusion of the autophagosome and lysosome (Figure 1). Overexpression of α-synuclein in human dopamine neurons led to an increase in autophagosomes and a decrease in autolysosomes. The fusion of autophagosomes and lysosomes relies on a complex of SNARE proteins. Researchers found that knocking down synaptosomal-associated protein 29 (SNAP29) had the same effect of α-synuclein overexpression and that SNAP29 co-expression in α-synuclein transduced-neurons partially restored autophagic flux and normalized the ratio of autophagosomes to autolysosomes^[144]^. Supporting these findings, SNAP29 was reduced in the substantia nigra of postmortem brain tissue from PD patients^[144]^. Further, interaction between ykt6, which plays a role in autolysosomal fusion, and SNAP29 is reduced in SNCA triplication iPS neurons pointing to a possible mechanism^[145]^.

Regarding fibrillar α-synuclein, a recent study focuses on how the structural and binding dynamics of α-synuclein fibrils can directly disrupt selective autophagy. Utilizing structural modeling, researchers predicted an α-synuclein fibrils-LC3B complex structure in which α-synuclein negatively charged C-terminal region interacts with the positively charged LC3B surface^[6]^. After resolving this key interaction, further structural analysis suggested that this specific fibril-LC3B binding site overlaps highly with the binding site for the p62 LC3-interacting region motif. This creates a possible competitive binding scenario in which an α-synuclein fibril could disrupt the LC3B-p62 interaction needed for p62-dependent selective autophagy (Figure 1). These studies confirmed that α-synuclein PFF treatment caused a reduction in p62-LC3B binding, thus suggesting a model where direct binding of α-synuclein fibrils to LC3B suppresses p62-mediated selective autophagy^[6]^.

ULK1 is involved in the initiation of autophagy. Interestingly, UKL1 mRNA and protein levels were decreased in microglia after treatment with α-synuclein PFFs, pointing to the possibility that fibrillar α-synuclein can disrupt the initiation of autophagy (Figure 1). Further, in seeking to understand how PFF treatment led to reduction in ULK1, researchers generated a knockdown of signal transducer and activator of transcription 1 (STAT-1), a transcription factor that downregulates ULK1 expression, and found increased ULK1 and LC3II and decreased p62^[146]^. Notably, they revealed enrichment of STAT1 at the Ulk1 promoter in PFF treated microglial cells as compared to control cells, pointing to a possible mechanism by which fibrillar α-synuclein reduces ULK1^[4]^.

An earlier study demonstrated that while LB-like α-synuclein inclusions induced by PFFs colocalize with autophagy machinery, they are not effectively degraded^[7]^. This study showed many LC3-II positive autophagosomes associated with α-synuclein aggregates, suggesting that PFF treatment may not prevent autophagosome formation, contradicting earlier evidence. Additionally, they demonstrated normal lysosomal function in PFF treated cells. Together, these findings point to a defect in autophagosome maturation. After autophagosome formation and prior to lysosomal fusion, an autophagosome fuses with multivesicular bodies (MVBs) to form a mature amphisome. Utilizing the MVB marker, CD63, they showed that any of the minimal LC3/CD63+ amphisomes present in PFF treated cells were not associated with α-synuclein aggregates and that any CD63 colocalization with α-synuclein aggregates was not LC3 positive, demonstrating an impairment at this maturation step (Figure 1)^[7]^. Interestingly, human α-synuclein transgenic flies show excess stabilization of the actin cytoskeleton, thus disrupting the proper F-actin dynamics required for autophagosome maturation^[147]^.

An additional point at which α-synuclein evades clearance that has garnered a lot of attention is lysosomal degradation. The majority of fibrils localize within lysosomes or at the lysosomal membrane and significantly increase the size and deform the organelles. Additionally, α-synuclein fibrils impair lysosome function as measured by activity of lysosomal proteases (Figure 1)^[148]^. Fibril treatment recruits galectin-3, a marker of lysosomal damage, and induces lysosome membrane permeabilization (LMP) in addition to seeding and aggregation of the soluble protein within lysosomes. Further, these α-synuclein-loaded lysosomes are redistributed to the cell periphery and have reduced degradative capacity. This peripheral distribution is correlated with TFEB translocation to the nucleus, suggesting its possible role in lysosome positioning. These lysosomes can be transferred to acceptor cells via TNTs, and the fibrils can induce LMP and seeding in the new cell, promoting the propagation of aggregates^[148,149]^. A key pathway in which α-synuclein disrupts lysosomal function is via reduction in activity of hydrolases, specifically within acidic compartments, including GCase and cathepsin B^[150]^. Exposure to both oligomeric and fibril α-synuclein reduces cathepsin D activity in H4 cells, highlighting impairment of lysosomal hydrolase function in both species^[151]^. This disruption may partially be due to the association of α-synuclein with vesicular fusion machinery. Specifically, researchers found that a mutant form of α-synuclein that is prone to aggregation caused diffuse localization of Rab1A, a regulator of ER-Golgi trafficking, and they were able to restore hydrolase activity and reduce α-synuclein accumulation by overexpressing Rab1A^[150]^. This points to the possibility of targeting protein trafficking to restore lysosome function in the context of α-synuclein pathology.

Ruptured lysosomes may undergo a selective autophagy process called lysophagy. During lysophagy, the damaged lysosome becomes ubiquitinated, leading to its eventual degradation by healthy lysosomes^[152]^. After ubiquitination, p62 is recruited to the damaged lysosome which further recruits HSP27 forming a platform for autophagosome biogenesis, leading to lysophagic clearance^[153]^. This process prevents leakage of contents, including α-synuclein aggregates. However, some lysosomes may be too severely damaged for lysophagic clearance. Interestingly, different fibril polymorphs vary in their toxicity to intracellular vesicles – twisted fibrils cause more damage than rod-like fibrils^[154]^. These findings highlight lysophagy’s protective role and suggest that enhancing this process could prevent α-synuclein fibril spread from damaged lysosomes. Further research should investigate which specific characteristics of fibril species contribute to their varying toxicity and propagation patterns.

LRRK2 variants contribute to common inherited forms of PD and are involved in vesicle trafficking and lysosomal stress response perhaps through phosphorylation of a subset of small Rab GTPases^[155]^. Multiple studies have shown that LRRK2 overexpression or the G2019S mutation accelerates α-synuclein pathology^[155–157]^. In the context of lysosomal overload stress, α-synuclein aggregates are released from microglia and macrophages that were preloaded with α-synuclein PFFs. This release is specifically mediated by LRRK2 phosphorylation of Rab10. Furthermore, internalized PFFs recruited LRRK2 and Rab8/Rab10 to the lysosomal surface, suggesting an increase in Rab phosphorylation at injured lysosomes resulting in the release of α-synuclein aggregates (Figure 1)^[134]^. Interestingly, the ATG8 conjugation system regulates the recruitment of LRRK2 and LC3 onto the single membrane of stressed lysosomes – a mechanism known as conjugation of ATG8 to single membranes (CASM), suggesting the involvement of non-canonical autophagy^[158]^.

In summary, α-synuclein can disrupt the autophagy process at multiple steps (Figure 1). The exact mechanism whereby α-synuclein aggregates or fibrils evade or disrupt the autophagy process requires more rigorous investigation in the future. Particularly, elucidation of the interplay between fibrillar α-synuclein and autophagy will enable the development of more precise therapeutic strategies for enhancing the clearance and preventing the spread of α-synuclein in synucleinopathies.

## Conclusion

In this review, we have discussed the intersection of the autophagy pathway with α-synuclein in the CNS. Under physiological conditions, α-synuclein is cleared through multiple pathways including the ubiquitin-proteasome system, chaperone-mediated autophagy, and macroautophagy, though aggregated forms cannot be degraded by the proteasome and instead impair this system^[87]^. Autophagy becomes increasingly important for maintaining α-synuclein homeostasis in aged neurons and for clearing oligomeric or modified protein species associated with pathological conditions. We elucidated a process of microglial selective autophagy of α-synuclein, termed “synucleinphagy”^[159]^. This study demonstrated that microglia take up neuron released α-synuclein leading to upregulation of the p62 receptor via the TLR4-NFkB pathway. Other studies have identified an additional receptor protein, NEMO, that aids in the recruitment of autophagic machinery^[103]^.

We also discussed the emerging evidence for how α-synuclein fibrils evade or disrupt the autophagy-lysosome pathway. The prion-like properties of α-synuclein to seed and self-propagate have been highly documented and established. Our discussion highlights the reported mechanisms by which α-synuclein is taken up and spreads across multiple cell types, thus presenting possible targets to halt intercellular transmission. The development of the preformed fibril model has been instrumental in demonstrating cell-to-cell transmission capabilities, with multiple receptors including LAG3, APLP1, and FAM171A2 facilitating uptake across various cell types^[107]^. Importantly, neurons, microglia, astrocytes, and oligodendrocytes all participate in this transmission network.

A critical finding is the intersection of autophagy dysfunction and unconventional secretion mechanisms. When canonical autophagy pathways are disrupted, α-synuclein is redirected toward pathogenic secretory routes including exophagy, lysosomal exocytosis, and alternative release mechanisms. Notably, while exosomal α-synuclein represents only a small fraction of total secreted protein, these vesicular forms demonstrate enhanced seeding capacity and correlate with disease severity^[130]^. A potential mechanism by which fibrillar α-synuclein can be degraded to some extent via autophagy was suggested through recruitment of the receptor proteins TBK1 and OPTN, as well as utilization of tunneling nanotubes to share the burden of degradation^[127,140]^.

Importantly, we have compiled the ways in which α-synuclein fibrils can evade or disrupt the autophagy lysosome pathway (ALP) from the initial binding to autophagy receptors and autophagy initiation to the formation of the autophagosomes, autophagosome maturation, autophagasome-lysosome fusion, and finally lysosomal degradation. Several promising therapeutic techniques are being studied to enhance autophagic clearance via activation of specific channels, targeted degradation by autophagosome-anchoring chimera peptides, and a variety of small-molecule inhibitors^[83,84,160,161]^. By identifying the precise points at which α-synuclein fibrils evade or disrupt ALP, we will be able to identify novel targets of ALP and develop better intervention strategies to halt the spread of synucleinopathies.

## Data Availability

Data sharing is not applicable to this article as no data were created or analyzed in this study.

## Abbreviations

ALP: Autophagy-lysosome pathway
APLP1: amyloid precursor-like protein 1
ATG: autophagy-related gene
CMA: Chaperon-mediated autophagy
CNS: Central Nervous System
DLB: Dementia with Lewy Bodies
EV: extracellular vesicle
FAM171A2: Family with sequence similarity 171
LAG3: Lymphocyte activation gene 3
LAMP: Lysosome-associated membrane protein
LB: Lewy body
LGALS3: galectin 3
LRRK2: Leucine rich repeat kinase 2
MSA: Multisystem Atrophy
MVB: multivesicular body
NEMO: nuclear factor-κB essential modulator
OPTN: Optineurin
PD: Parkinson’s disease
PFF: Pre-formed fibril
PTM: Post-translational modification
SAA: Seed amplification assay
SN: Substantia nigra
SNAP29: synaptosomal-associated protein 29
SNARE: SNAP receptor
SQSTM1: Sequestosome 1
STAT1: signal transducer and activator of transcription 1
SUMO: Small ubiquitin-like modifier
TBK1: Tank-binding kinase 1
TLR: Toll like receptor
TNT: tunneling nanotube
ULK: unc-51 like autophagy activing kinase
UPS: Ubiquitin-proteasome system
